# NDUFA11 may be the disulfidptosis-related biomarker of ischemic stroke based on integrated bioinformatics, clinical samples, and experimental analyses

**DOI:** 10.3389/fnins.2024.1505493

**Published:** 2025-01-14

**Authors:** Sijun Li, Ningyuan Chen, Junrui He, Xibao Luo, Wei Lin

**Affiliations:** ^1^Department of Geriatric Rehabilitation, Jiangbin Hospital, Nanning, China; ^2^Department of Pathophysiology, Guangxi Medical University, Nanning, China

**Keywords:** ischemic stroke, disulfidptosis-related biomarkers, machine-learning model, NDUFA11, protein complexes

## Abstract

**Background:**

Programmed cell death plays an important role in neuronal injury and death after ischemic stroke (IS), leading to cellular glucose deficiency. Glucose deficiency can cause abnormal accumulation of cytotoxic disulfides, resulting in disulfidptosis. Ferroptosis, apoptosis, necroptosis, and autophagy inhibitors cannot inhibit this novel programmed cell death mechanism. Nevertheless, the potential mechanisms of disulfidptosis in IS remain unclear.

**Methods:**

The GSE16561 dataset was used to screen for differentially expressed disulfidptosis-related biomarkers (DE-DRBs). A correlation between the DE-DRBs was detected. The optimal machine-learning (ML) model and predictor molecules were determined. The GSE58294 dataset was used to verify the accuracy of the optimal ML model. The DE-DRB expression was detected in the blood of patients with IS. Based on IS models, experimental analyses were performed to verify DE-DRB expression and the correlation between DE-DRBs.

**Results:**

Leucine-rich pentatricopeptide repeat-containing (LRPPRC) and NADH dehydrogenase [ubiquinone] 1 alpha subcomplex subunit 11 (NDUFA11) were identified as DE-DRBs. The NADH: ubiquinone oxidoreductase core subunit S1 (NDUFS1) interacted with NDUFA11 and LRPPRC. The support vector machine (SVM) model was identified as the optimal ML model. The NDUFA11 expression level in the blood of patients with IS was 20.9% compared to that in normal controls. NDUFA11 expression was downregulated in the *in vitro/in vivo* models of IS. The number of formed complexes of NDUFS1 and NDUFA11 decreased in the *in vitro/in vivo* models of IS.

**Conclusion:**

This research suggests that NDUFA11 is a specific DRB for IS and demonstrates alterations in the disulfidptosis-related protein complexes NDUFS1-NDUFA11.

## Introduction

Stroke is the second leading cause of disability and death worldwide and imposes a heavy burden on individuals and society (Feigin et al., 2019). Stroke is classified into ischemic stroke (IS) and hemorrhagic stroke. IS is the most common subtype, accounting for >60% of all stroke cases (Ge R. et al., 2024). IS affects 11.6 million people, 63% of whom live in low- and middle-income countries (Saini et al., 2021). Cerebral artery blockage causes ischemia and hypoxia in the corresponding brain areas, leading to neuronal death, which is the primary cause of disability and death in IS (Liu M. et al., 2020; Staessens and De Meyer, 2020). Initially, reversible loss of tissue function occurs due to insufficient blood supply to the brain tissue. Infarcts with a loss of neurons and supporting structures occur with prolonged ischemia (Feske, 2021). After cerebral ischemia, neurons lose their electrical function, leading to membrane dysfunction caused by calcium inflow, calcium-dependent excitatory toxicity, generation of reactive oxygen species (ROS), cell membrane rupture, and cell lysis (Feske, 2021). Neuronal damage can cause complete central nervous system dysfunction and affect other tissues and organs, resulting in serious consequences (Levinthal and Strick, 2020; Patel et al., 2022; Qin et al., 2024; Shao and Li, 2023; Wang et al., 2018; Wang et al., 2021).

Programmed cell death plays an important role in neuronal injury and cell death after IS (Tuo et al., 2021). Currently, the primary mechanisms of programmed cell death include apoptosis, pyroptosis, necrosis, autophagy, ferroptosis, and cuproptosis (Cobine and Brady, 2022; Fricker et al., 2018; Li et al., 2022; Nisa et al., 2022). These mechanisms are involved in the pathogenesis of IS (Ajoolabady et al., 2021; Cui et al., 2021; Liu et al., 2023b; Luo et al., 2022; Wang et al., 2023). Recent studies have revealed that glucose deficiency causes programmed cell death. Glucose deficiency can cause abnormal accumulation of cytotoxic disulfides, including cystine, thereby leading to disulfide stress and programmed cell death (Joly et al., 2020; Liu X. et al., 2020; Liu et al., 2023a). This is a novel type of programmed cell death mechanism known as disulfidroptosis. Common cell death inhibitors, including ferroptosis, apoptosis, necroptosis, and autophagy inhibitors, cannot inhibit disulfidptosis (Liu et al., 2023a). Under normal physiological conditions, the reduced form of nicotinamide adenine dinucleotide phosphate (NADPH) produced by glucose is sufficient to counteract the toxic effects of disulfide stress on cells. When glucose is insufficient, the biological processes of over-uptake of cystine and reduction of cystine to cysteine deplete the NADPH pool, resulting in NADP/NADPH imbalance. Under these circumstances, actin increases cell sensitivity to disulfide stress, ultimately leading to massive accumulation of disulfide molecules and rapid cell death (Joly et al., 2020; Koppula et al., 2017; Liu X. et al., 2020; Liu et al., 2023a). Disulfidptosis may specifically affect mitochondrial function by disturbing mitochondrial genes, particularly in neurons (Ge H. et al., 2024). Disulfidptosis also functions in other diseases, including pan-cancer, lung adenocarcinoma and Alzheimer’s disease (Huang et al., 2023; Huang et al., 2024; Liu and Tang, 2023).

Glucose deficiency and mitochondrial dysfunction are common pathological characteristics of IS (Sifat et al., 2022) and can cause neuronal death (Li et al., 2023d). IS may also cause abnormal metabolism in cystine (Fan et al., 2023). Previous studies have demonstrated that disulfidptosis may also be involved in IS pathogenesis (Liu et al., 2024; Qin et al., 2023; Zhao et al., 2024). Liu et al. (2024) suggested that PRDX1 plays a protective role in stroke by inhibiting disulfidptosis. Qin et al. (2023) used bioinformatics methods to analyze single-cell data from public databases and found the relationship between disulfidptosis and IS, as well as therapeutic targets. Zhao et al. (2024) found that disulfidptosis-related biomarker (DRB) SLC7A11 in human microglia could improve M2 polarization and inhibit M1 polarization in an *in vitro* model of IS. Nevertheless, these studies did not integrate bioinformatics, clinical samples, and *in vitro* and *in vivo* models to analyze the potential mechanism of disulfidptosis in IS. Identifying reliable biomarkers and investigating the potential mechanisms of disulfidptosis in IS are critical for predicting and treating IS. We identified differentially expressed disulfidptosis-related biomarkers (DE-DRBs) using bioinformatics, clinical samples, and *in vitro* and *in vitro* models of IS. We elucidated the potential mechanism of disulfidptosis by validating protein-protein interaction (PPI).

## Materials and methods

### Data acquisition and pre-processing

Screening for biomarkers from blood samples is easier and less damaging to patients than from cerebrospinal fluid and brain tissue. Considering the use of clinical samples for validation, all datasets were derived from blood samples. Datasets (GSE16561 and GSE58294) were downloaded from the Gene Expression Omnibus repository.^1^ The GSE16561 (63 blood samples), including 24 healthy control individuals (Ctrl group) and 39 individuals with IS, was used as a training dataset to screen DE-DRBs and build machine-learning (ML) models. The GSE58294 (92 blood samples), including 23 healthy individuals and 69 individuals with IS, was used as a testing dataset to verify the accuracy of the ML model. The Perl programming language was used to pre-process the two datasets. Ten DRBs were referred to by Liu et al. (2023a). Liu et al. (2023a) conducted a whole-genome CRISPR-Cas9 screening in SLC7A11-overexpressing 786-O cells under glucose-replete and -starved conditions and the relative fold changes for guide RNAs between these two conditions were analyzed and presented as a normZ score plot. Based on these methods, Liu et al. (2023a) obtained 10 DRBs, including *GYS1, NDUFS1, OXSM, LRPPRC, NDUFA11, NUBPL, NCKAP1, RPN1, SLC3A2*, and *SLC7A11*. The R Programming Language (version 4.3.2) was used to analyze the data.

### Identification of DE-DRBs in IS

Two R packages (“limma” and “ggpubr”) were used to screen and visualize DE-DRBs. The “RCircos” R package was used to exhibit the position of DE-DRBs on chromosomes. The “corrplot” package was used to correlate DE-DRBs and explore the correlation between DE-DRBs. Interactions between proteins are essential for cellular function. Due to the limitations of experimental techniques, computational tools were used to analyze PPI (Han et al., 2020). Researchers have gained valuable data to establish advanced databases for analyzing and predicting protein-protein networks by effectively integrating experimental results and computational techniques (Szklarczyk et al., 2023). The PPI analysis of all DRMs was performed using STRING.^2^

### Clustering patients with IS

Based on the DE-DRBs, patients with IS in the GSE16561 dataset were divided into subtypes using the “ConsensusClusterPlus” R package. Cluster analysis is an integral part of precision medicine and systems biology, which defines groups of patients or biomolecules. Consensus clustering is an ensemble approach that is widely used in these areas, combining the output from multiple runs of a non-deterministic clustering algorithm (Coleman et al., 2022). The k value was defined as 1–9, and different subtypes were generated. Based on the highest intra-group correlations, lowest intra-group correlations, and highest concordance score, the optimum k value and number of clusters were determined. Subtypes were visualized using principal component analysis (PCA).

### Gene set variation analysis (GSVA)

According to Li et al. (2023b) GSVA enrichment analysis was employed to clarify the enriched gene sets for DE-DRB clusters using the “GSVA” R package (*p* < 0.05, considered a significant change). The “c5.go.symbols” and “c2.cp.kegg.symbols” were obtained from the molecular signatures database^3^ for GSVA analysis.

### Weighted gene co-expression network analysis (WGCNA)

According to Li et al. (2023b) a WGCNA identification co-expression module was constructed using the “WGCNA” R package. The colors were randomly matched to the module. The overall gene expression profile of the module was represented by module signature genes. Each module displayed a correlation coefficient and a *p*-value. The module with the highest correlation coefficient and the lowest *p*-value was considered the most significant. Module significance represents the relationship between modules and disease states. Gene significance was defined as the correlation between a gene and the clinical phenotype. Disease-WGCNA was constructed based on Ctrl and IS groups. Cluster-WGCNA was constructed based on the different clusters of patients with IS. Genes derived from the module that exhibited the most significant relationship with IS were selected. Genes derived from module modules closely related to IS clustering were selected. The intersecting genes for these two modules were obtained using the “Venn” R package.

### Construction and validation of predictive and nomogram models

Based on the intersection genes derived from WGCNA modules, the “caret” R package was used to construct the ML models, including random forest (RF), support vector machine (SVM), generalized linear model (GLM), and eXtreme gradient boosting (XGB). The “pROC” R package was used to visualize the area below the receiver operating characteristic (ROC) curve. The ROC curve was used to estimate the discriminatory performance of a novel diagnostic test, identify the optimal cut-off value for a test that maximizes sensitivity and specificity, and evaluate the predictive value of a certain biomarker or risk, prediction score (Roumeliotis et al., 2024). The model with the largest area under the ROC curve (AUC) and the smallest residual value was considered the optimal ML model. Three important variables were selected as key predictive genes related to IS. Based on the key prediction genes for IS, ROC curve analysis was conducted on patients with IS in the GSE58294 dataset, and the accuracy of the diagnostic model was verified. Based on the three important variables of the optimal ML model, a nomogram model of IS was constructed using the “rms” R package to evaluate the IS cluster.

### Verifying the mRNA expression of DE-DRBs in patients with IS

Six blood samples from healthy individuals and six from patients with IS were collected to identify DE-DRGs. The diagnostic criteria for IS were based on previous guideline (European Stroke Organisation Executive Committee, and ESOW Writing Committee., 2008). The exclusion criteria were as follows: Hemorrhagic stroke, malignant tumor, metabolic disorders, infectious disease, pneumonia, and severe impairment of consciousness. Informed consent was obtained from patients or their guardians. This study adhered to the principles of the Declaration of Helsinki. Ethical approval was granted by the Institutional Review Board of Jiangbin Hospital (No. KY-2024YJS-010).

The primers for DE-DRGs were as follows: Leucine-rich pentatricopeptide repeat-containing (LRPPRC) forward primer: AATGGCGCAGCTTTAAGAGG, reverse primer: ATGTTGGTGGATGGTTCTGC; NADH dehydrogenase [ubiquinone] 1 alpha subcomplex subunit 11 (NDUFA11) forward primer: CGCTGCCTACAGAGTCACA, reverse primer: GCCACCGAGGAAGTAGTTCA. Human endogenous reference gene GAPDH (B661104; Sangon Biotech). RNAiso Blood (9113; Takara) was used to obtain total RNA from the blood. The HiScript III 1st Strand cDNA Synthesis Kit (R312-01, Vazyme) was used to obtain cDNA from the total RNA. Taq Pro Universal SYBR qPCR Master Mix (Q712-02, Biosharp) and a 7500 Real-Time PCR System (Applied Biosystems) were used to perform RT-PCR.

### Construction of the *in vitro* model of IS

The oxygen-glucose deprivation/reperfusion (OGD/R) model is considered an *in vitro* model of IS (Lai et al., 2020; Zhu et al., 2021). An *in vitro* OGD/R model was constructed by culturing neurons from the cortex of 24-h-old Sprague-Dawley (SD) rats. The details of the neuron culture were referenced in our previous study (Li et al., 2023c). Before culturing, the Petri dishes were incubated with poly-D-lysine (Gibco, A3890401) at 37°C for 10 h. Then, two media were prepared: Serum-neurobasal medium containing 88% Dulbecco’s Modified Eagle Medium/F-12(DMEM/F12, Gibco, A4192001), 1% Glutamax (Gibco, 35050061), 1% penicillin-streptomycin (Gibco, 15140-122), and 10% serum (Gibco, A3160902); serum-free neurobasal medium included 96% Neurobasal™-A (Gibco, 10888022), 1% Glutamax (Gibco, 35050061) + 1% penicillin-streptomycin (Gibco, 15,140-122), and 2% B27 supplement (Gibco, 17504044). The cortex was dissected in ice-cold Hank’s buffered salt solution (Thermo Fisher, #14185052) with 10 mM N-2-hydroxyethylpiperazine-N-2-ethane sulfonic acid (HEPES). The cortex was snipped and incubated with 0.125% trypsin-EDTA (Gibco, A4192001) at 37°C for 12 min. After trypsin digestion was terminated using the serum-neurobasal medium, non-dissociated tissue was removed by filtering through a 40-mm nylon mesh strainer. Cortex neurons were plated in dishes containing serum-neurobasal medium (cell density: 160/mm^2^). After 10 h, the serum-neurobasal medium was removed and replaced with a serum-free neurobasal medium. Half of the medium was replaced with serum-free neurobasal medium on day four or once a week. After 8 days *in vitro*, the neurons were divided into the control and OGD/R groups. The specific method is as follows (Lai et al., 2020). Neurons in the control group were cultured normally in a complete culture medium. Neurons in the OGD/R group were washed three times with phosphate-buffered saline and placed in an anaerobic chamber containing 5% CO_2_, 95% N_2_, and glucose-free Dulbecco’s modified Eagle medium (DMEM, Gibco, NY, United States, Cat# 11966-025) at 37°C for 2 h. Then, the culture medium was reverted to the normal medium, and the neurons were maintained in a 5% CO_2_ incubator at 37°C for 12 h to induce re-oxygenation injury.

### Construction of the *in vivo* model of IS

The middle cerebral artery occlusion (MCAO) model is considered an *in vivo* model of IS (Lai et al., 2020; Zhu et al., 2021). Sixteen SD rats were used for animal experiments. Immunofluorescence (IF) experiments were performed on two rats to determine the localization of DE-DRBs. Fourteen SD rats were divided into sham and MCAO groups, with seven in each group. All rats were anesthetized and skinned, and their neck skin was disinfected to isolate the carotid artery. The common and internal carotid arteries were temporarily clipped using microvascular clips in the MCAO group. A nylon filament (MEYUE, M8509) was advanced from the right external carotid artery to the internal carotid artery until it reached the root of the middle cerebral artery. The rats in the sham group underwent the same surgery without microvascular clips and filaments. After surgery, all rats were transferred to an electric blanket to maintain their warmth. Neurological impairment in rats was evaluated after recovery. The evaluation criteria for functional impairment were as follows: Score 0, no sign of nerve damage; score 1, incomplete extension of the forelimb; score 2, circle to the left; score 3, fall to the left; score 4, unconscious loss of voluntary walking. The rats underwent the next experiment 7 days after the operation.

### Triphenyl tetrazolium chloride (TTC) staining

After anesthesia, the rats were sacrificed, and two rats from each group were randomly selected for TTC staining. The brain tissue was collected and placed directly into the optimal cutting temperature compound embedding agent for quick freezing at −20°C for 30 min. Then, the brain was sliced, and each slice was approximately 2–3 mm thick. The TTC solution was preheated to 37°C. Then, the slices were incubated with TTC solution at 37°C for 30 min in the dark. The tissues were gently shaken for 10 min to ensure equal exposure to the dye solution, and the staining results were observed.

### IF staining

The localization of DE-DRBs was observed using IF staining to determine whether DE-DRBs were expressed in neurons and rat cortex *in vitro*. Specific methods can be found in the study by Li et al. (2023c). The primary antibodies were anti-LRPPRC antibody (Abcam, ab259927, 1:200) and anti-NDUFA11 antibody (Abclonal, A16239, 1:100) (Yang et al., 2022). A microscope (Olympus BX53) was used to observe neurons and obtain images. A high-resolution slide scanning system (3DHISTECH Ltd., Pannoramic MIDI) was used to observe paraffin sections and obtain images. CaseViewer (version 2.4) was used to obtain images of the paraffin sections.

### Western blot

A protein extraction kit (Invent Biotechnologies, SD-001/SN-002) was used to extract proteins. Western blotting was performed as in our previous research (Li et al., 2023c). The primary antibodies were anti-LRPPRC antibody (Abcam, ab259927, 1:1000), anti-NDUFA11 antibody (Abclonal, A16239, 1:1000) (Yang et al., 2022), and Anti-Glyceraldehyde-3-phosphate dehydrogenase (GAPDH) antibody (Abcam, ab8245, 1:10000). The Odyssey Infrared Imaging System (LI-COR Biosciences) was used to obtain experimental results. All target proteins were normalized to the internal reference protein GAPDH using the ImageJ software.

### Co-immunoprecipitation (Co-IP)

Natural proteins were extracted using a protein extraction kit (Invent Biotechnologies, SD-001/SN-002) (Hendrix et al., 2021). An immunoprecipitation kit (Sangon Biotech, C600689) was used to perform Co-IP (Li et al., 2023c). The natural total protein was incubated with anti-NADH: Ubiquinone oxidoreductase core subunit S1 (NDUFS1) antibody overnight at 4°C to generate antigen-antibody complexes. The antigen-antibody complex was incubated with A/G + agarose protein at 4°C overnight. Finally, Western blot analysis was performed. The primary antibodies were anti-LRPPRC antibody (Abcam, ab259927, 1:1000) and anti-NDUFA11 antibody (Abclonal, A16239, 1:1000) (Yang et al., 2022). The Odyssey Infrared Imaging System (LI-COR Biosciences) was used to obtain experimental results. The ImageJ software was used to analyze the results. The pull-down protein levels obtained by Co-IP were normalized using IP protein bands of NDUFS1. The levels of co-precipitated proteins in each group were compared.

### Network pharmacological analysis

A network pharmacological analysis was performed to investigate the therapeutic strategies further. Gene and drug information were downloaded from the Drug SlGnatures DataBase.^4^ The R packages “clusterProfiler,” “org.Hs.eg.db,” “enrichplot,” “tidytable,” and “ggplot2” were used to perform the network pharmacological analysis. The pharmacological network was visualized using the Cytoscape software (version 3.8.0).

### Statistical analyses

The mean values of the two groups were compared using an independent sample *t*-test. The results are expressed as mean ± standard deviation. The Statistical Package for the Social Sciences software (version 25.0) was used to perform an independent sample *t*-test. The *p-*value < 0.05 was considered statistically significant.

## Results

### Downregulated expression of two DRBs in IS

**FIGURE 1:**
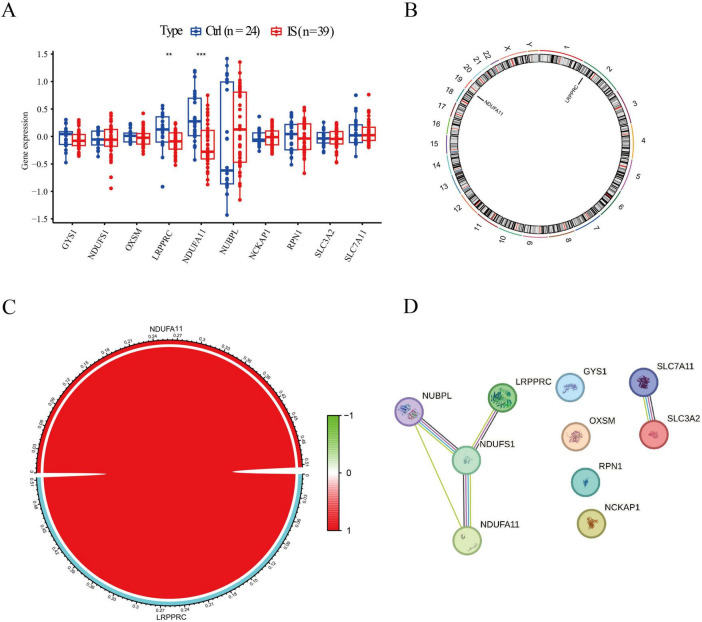
Identification of DE-DRBs in patients with IS. **(A)** Two genes were identified as DE-DRBs. The LRPPRC and NDUFA11 expression was decreased in the IS group (*n* = **p < 0.01, ***p < 0.001). **(B)** Locations of the two DE-DRBs on chromosomes. **(C)** Correlation analysis demonstrates a significant positive correlation between LRPPRC and NDUFA11 (correlation coefficient = 0.513). **(D)** PPI analysis suggested that NDUFS1 interacts with NDUFA11, NUBPL, and LRPPRC. DE-DRBs, differentially expressed disulfidptosis-related biomarkers; LRPPRC, leucine-rich pentatricopeptide repeat-containing; NDUFA11, NADH dehydrogenase [ubiquinone] 1 alpha subcomplex subunit 11; IS, ischemic stroke; PPI, protein-protein interaction.

Initially, we evaluated the expression profiles of DRBs in control and IS groups to elucidate their biological functions in IS occurrence and development. We identified two genes as DE-DRBs. The LRPPRC versus Ctrl (*p* < 0.01) and NDUFA11 versus Ctrl (*p* < 0.001) expression decreased in the IS group (Figure 1A). Figure 1B presents the locations of the two DE-DRBs on chromosomes. Correlation analysis revealed a significant positive correlation between LRPPRC and NDUFA11 expression in the IS group (correlation coefficient = 0.513; Figure 1C). Then, We performed PPI analysis on these two DE-DRBs, but there is no interaction between them, so we performed PPI analysis on 10 DRBs. Subsequently, PPI analysis suggested that NDUFS1 interacted with NDUFA11, NUBPL, and LRPPRC (Figure 1D), indicating that NDUFA11 was associated with LRPPRC through NDUFS1. NDUFS1 forms protein complex with NDUFA11, LRPPRC, NUBPL.

### Two DRBs-clusters were distinguished in IS

**FIGURE 2:**
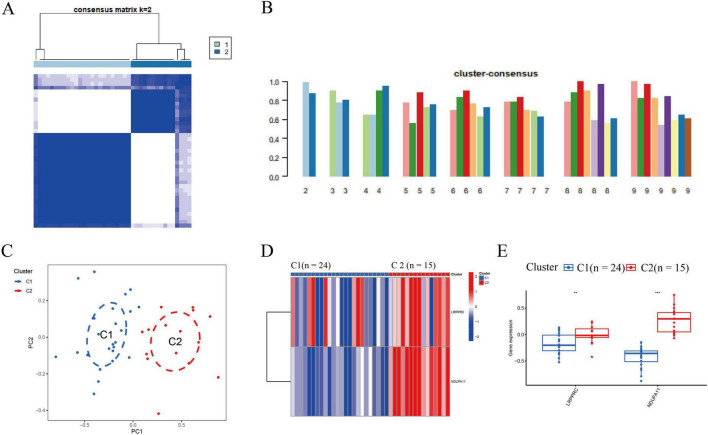
Identification of molecular subtypes associated with disulfidptosis in IS. **(A)** The cluster number was the most stable when *k* = 2. **(B)** Cluster diagram for subtype analysis of IS samples. The intra-group correlations were the highest, whereas the inter-group correlations were lowest when *k* = 2. The concordance score for each subtype was highest when *k* = 2. **(C)** The PCA reveals significant differences between the two clusters. Thirty-nine patients with IS were divided into clusters 1 (*n* = 24) and 2 (*n* = 15), which were significantly different. **(D)** Distinct DRB expression profiles were observed between clusters 1 and 2. **(E)** Boxplot presented that the LRPPRC and NDUFA11 expression decreased in cluster 1 (**p < 0.01, ***p < 0.001). IS, ischemic stroke; DRB, disulfidptosis-related biomarkers; LRPPRC, leucine-rich pentatricopeptide repeat-containing; NDUFA11, NADH dehydrogenase [ubiquinone] 1 alpha subcomplex subunit 11.

Based on the expression profiles of two DE-DRMs, 39 patients with IS were classified into different subtypes using a consensus clustering algorithm. The number of clusters was most stable when *k* = 2 (Figure 2A). The consistency score of each subtype was the highest when *k* = 2 (Figure 2B). According to PCA analysis, 39 patients with IS were divided into clusters 1 and 2 (*n* = 24 and *n* = 15, respectively; Figure 2C). The differences in DRMs between clusters 1 and 2 were comprehensively evaluated to explore the molecular characteristics of the two clusters. The LRPPRC and NDUFA11 expression levels were lower in cluster 1 than in cluster 2 (Figures 2D, E).

### Biological functions and pathway activities in IS

**FIGURE 3:**
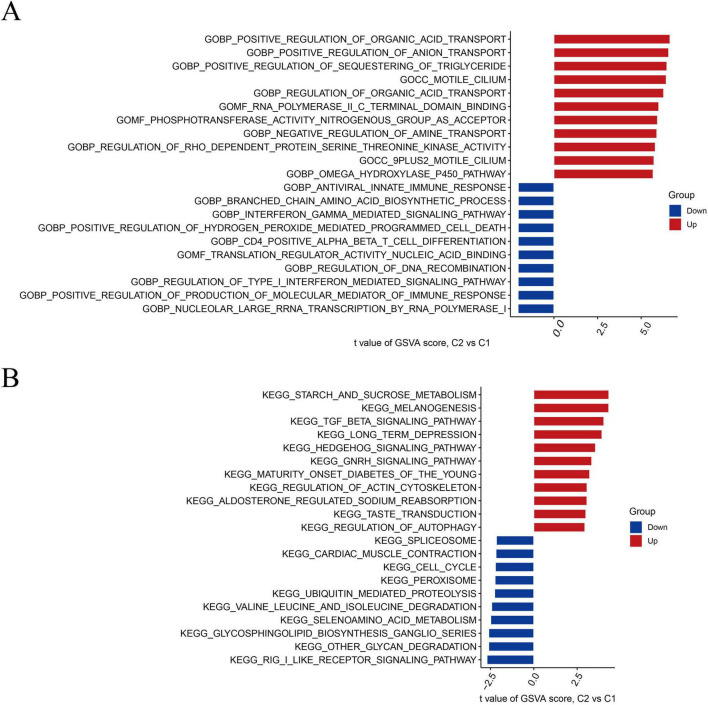
Biological functions and pathway activities of the two DRB clusters. **(A)** Differences in biological functions between clusters 1 and 2. **(B)** Differences in hallmark pathway activities between clusters 1 and 2, with samples ranked using the *t*-value of the GSVA method. DRB, disulfidptosis-related biomarkers; GSVA, gene set variation analysis.

Based on GSVA analysis, gene ontology (GO) results displayed that positive regulation of organic acid transport, anion transport, sequestering of triglycerides, and aldosterone-regulated sodium reabsorption were active in cluster 2. Positive regulation of the production of molecular mediators of the immune response, type-I interferon-mediated signaling pathway, and nucleolar larger RNA transcription by RNA polymerase I was active in cluster 1 (Figure 3A). The Kyoto Encyclopedia genes and genomes (KEGG) results revealed that starch and sucrose metabolism, melanogenesis, and tumor growth factor (TGF-β) signaling pathways were significantly enriched in cluster 2. The spliceosome, cardiac muscle contraction, and cell cycle were significantly enriched in cluster 1 (Figure 3B).

### Ten genes were closely associated with IS

**FIGURE 4:**
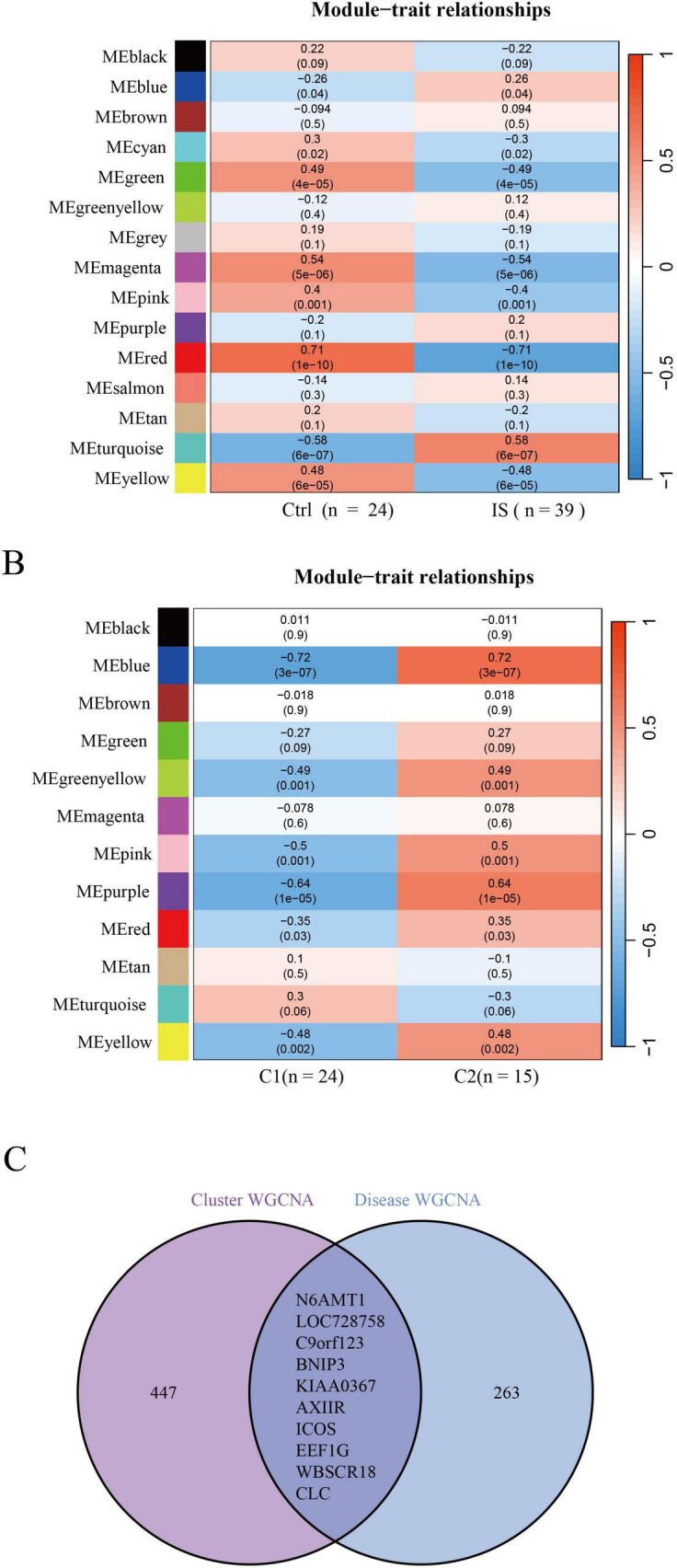
Identification of gene modules and co-expression networks associated with IS. **(A)** Disease-WGCNA: Genes in the red module exhibited the most significant relationship with IS. **(B)** Cluster-WGCNA: module-clinical feature relationship analysis demonstrated a high correlation between the blue module and IS clusters. **(C)** Identification of the intersecting genes of disease- and cluster-WGCNA. The intersection of genes in the two modules yielded 10 genes. IS, ischemic stroke; WGCNA, weighted gene co-expression network analysis.

A total of 273 genes in the red module exhibited the strongest relationship with IS (Figure 4A). Additionally, based on DE-DRBs clustering, we performed a relationship analysis of key gene modules (clusters 1 and 2) closely related to clinical features. We found 457 genes in the blue module highly correlated with the IS cluster (Figure 4B). Based on disease- and cluster-WGCNA, we obtained two modules with 10 overlapping genes (*N6AMT1, LOC728758, C9orf123, BNIP3, KIAA0367, AXIIR, ICOS, EEF1G, WBSCR18*, and *CLC*) that may be closely associated with IS (Figure 4C).

### The SVM model was the best ML model

**FIGURE 5:**
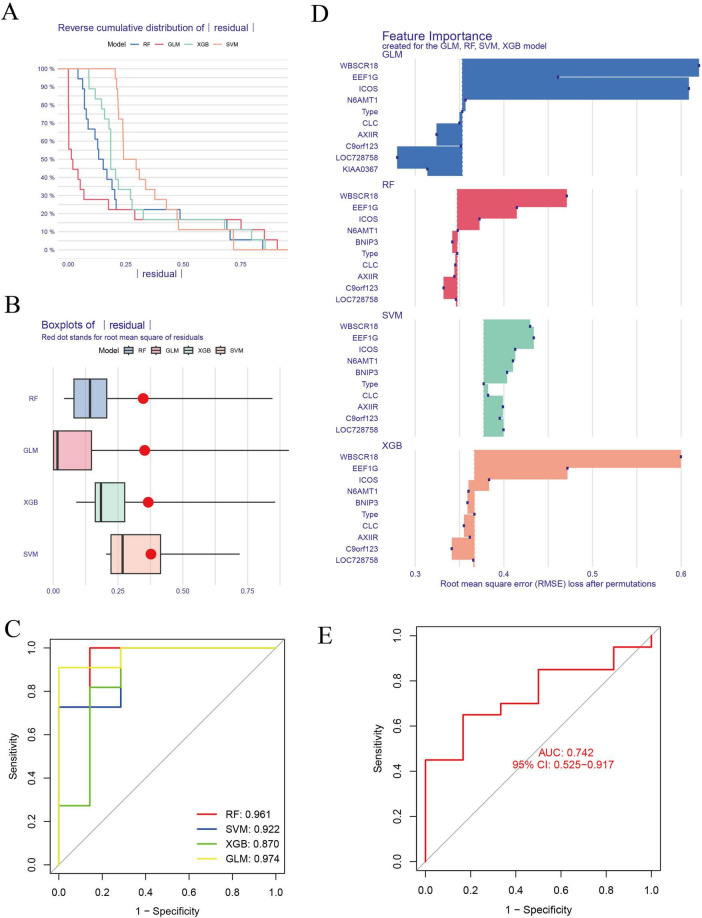
Construction and evaluation of ML models for predicting IS. **(A,B)** Residuals of the four models are presented. **(C)** The AUC for each model is as follows: RF, AUC = 0.961; SVM, AUC = 0. 922; XGB, AUC = 0.974; GLM, AUC = 0.870. **(D)** Genes for the top 15 important features of each model were sequenced based on RMSE. **(E)** The SVM model was the best ML model, with an AUC of 0.742. ML, machine-learning; ROC, receiver operating characteristic; AUC, the area under the ROC; RF, random forest; SVM, support vector machine; GLM, generalized linear model; XGB, eXtreme gradient boosting.

We screened these 10 overlapping genes using ML models to identify the best disease-predicting genes. Figures 5A, B illustrate the residuals of the four models. Then, we assessed the discriminant performance of the four ML algorithms by calculating their ROC curves (Figure 5C). The AUC values for all models were as follows: AUC_RF_ = 0.961; AUC _*SVM*_ = 0.922; AUC_*XGB*_ = 0.974; AUC _*GLM*_ = 0.870. Based on root-mean-square error (RMSE), the top 15 important genes in each model were sequenced (Figure 5D). The AUC values of RF, SVM, and XGB were >0.9, and the testing dataset was used to verify these three models. The SVM model was the best ML model, with an AUC of 0.742 (Figure 5E). Finally, the three most important genes (BNIP3, N6AMT1, and ICOS) in the SVM model were considered predictors of IS risk.

### *BNIP3, N6AMT1*, and *ICOS* could be used as predictors of IS

**FIGURE 6:**
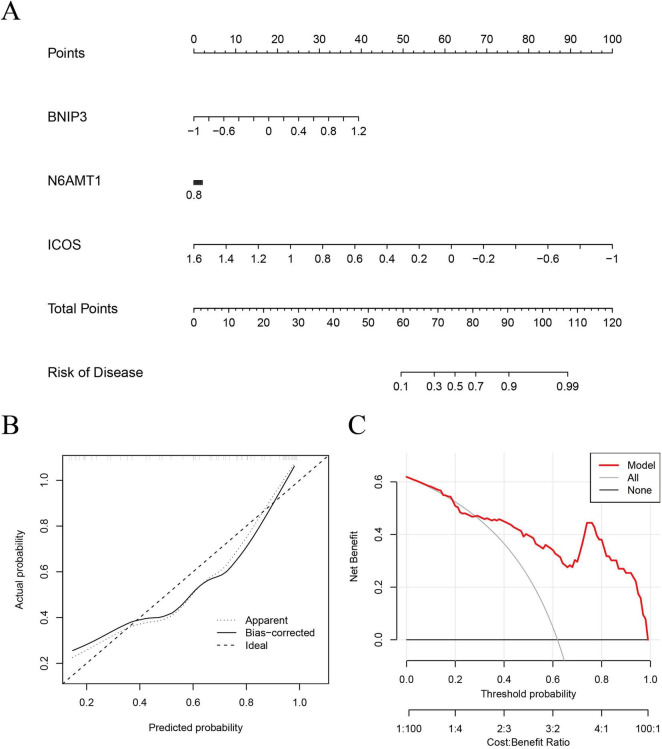
Validation of an ML model based on five genes for predicting IS. **(A)** A nomogram model was constructed to estimate the risk of disulfidptosis clusters in IS. **(B)** Construction of the calibration curve: Calibration curve analysis exhibited that the solid line was near the dotted line, suggesting relatively high accuracy of the nomogram. **(C)** Construction of the decision curve: DCA exhibited that the red line moved away from the gray line, suggesting relatively high accuracy of the nomogram. ML, machine-learning; DCA, decision curve analysis.

Based on the three most important genes (*BNIP3, N6AMT1*, and *ICOS*) in the SVM model, a nomogram model was constructed to evaluate IS risk (Figure 6A). Correction curve and decision curve analysis (DCA) were used to evaluate the prediction efficiency of the nomogram model. According to the calibration curve, the error between the actual and predicted risks for the IS cluster is small (Figure 6B). The DCA results suggested that the nomogram exhibited good accuracy (Figure 6C).

### NDUFA11 expression and the number of NDUFS1-NDUFA11 protein complexes were decreased in IS

**FIGURE 7:**
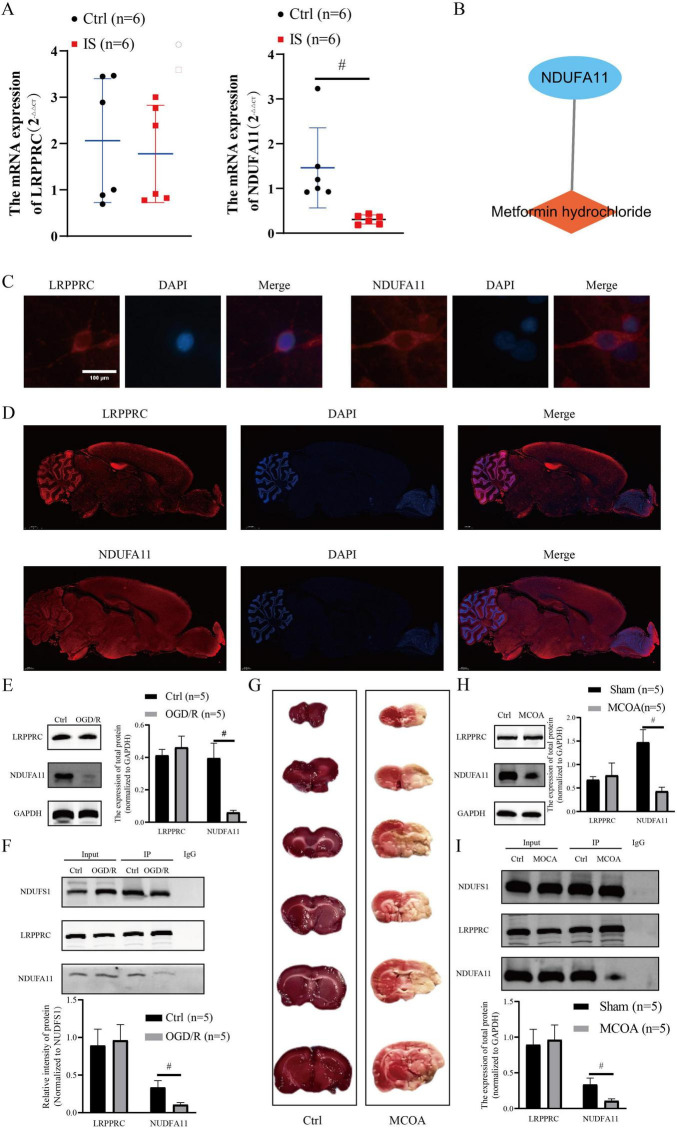
Verification of DE-DRB expression in clinical samples and *in vitro/in vivo* models. **(A)** The NDUFA11 expression was significantly reduced in the blood of patients with IS (*n* = 6 in each group versus control; independent samples *t*-test; #*p* < 0.01). **(B)** Metformin hydrochloride may be a potential target drug for NDUFA11. **(C)** The DE-DRMs are expressed in neurons (×400, scale: 100). **(D)** DE-DRMs expressed in the cortex (×200, scale: 2000 μm). **(E)** The NDUFA11 expression decreased significantly (*n* = 5 in each group versus control; independent samples *t*-test; #*p* < 0.01). **(F)** The NDUFS1-NDUFA11 protein complex decreased significantly in the OGD/R group (*n* = 5 in each group versus control; independent samples *t*-test; #*p* < 0.01). **(G)** The TTC results indicate significant ischemic areas in the right cerebral hemisphere of the MCAO group. **(H)** The NDUFA11 expression decreased significantly (*n* = 5 in each group versus control; independent samples *t*-test; #*p* < 0.01). **(I)** The NDUFS1-NDUFA11 protein complex decreased significantly in the MCAO group (*n* = 5 in each group versus control; independent samples t-test; #*p* < 0.01). DE-DRBs, differentially expressed disulfidptosis-related biomarkers; IS, ischemic stroke; LRPPRC, leucine-rich pentatricopeptide repeat-containing; NDUFA11, NADH dehydrogenase [ubiquinone] 1 alpha subcomplex subunit 11; NDUFS1, NADH: ubiquinone oxidoreductase core subunit S1; OGD/R, oxygen-glucose deprivation/reperfusion; MCAO, middle cerebral artery occlusion.

The LRPPRC expression did not change significantly, while NDUFA11 expression reduced significantly in the blood of patients with IS. The NDUFA11 expression level in the blood of patients with IS was 20.9% compared to that in normal controls (*p* < 0.01; Figure 7A). Network pharmacological analysis revealed that metformin hydrochloride might be a target drug for NDUFA11 (Figure 7B). The IF results displayed that DE-DRMs were expressed in neurons and cortex (Figures 7C, D). Subsequently, OGD/R models were constructed to detect DE-DRM expression. The results revealed that LRPPRC expression did not change significantly in the OGD/R model group, while NDUFA11 reduced significantly (*p* < 0.01; Figure 7E). Based on PPI results, the relationship between NDUFS1 and the two DE-DRBs forming protein complexes was analyzed using a Co-IP assay. The results indicated that the NDUFS1-LRPPRC protein complex did not change significantly, while the NDUFS1-NDUFA11 protein complex decreased significantly in the OGD/R group (*p* < 0.01; Figure 7F). Then, the MCAO model was constructed, and TTC results revealed significant ischemic areas in the right cerebral hemisphere in the MCAO group (Figure 7G). Western blot results showed that LRPPRC expression did not change significantly in the MCAO group, while NDUFA11 expression decreased significantly (*p* < 0.01; Figure 7H). Co-IP assay results indicated that the NDUFS1-LRPPRC protein complex did not change significantly, while the NDUFS1-NDUFA11 protein complex decreased significantly (*p* < 0.01; Figure 7I).

## Discussion

Neurons have non-regenerative properties, and damage to the central nervous system caused by dead neurons is almost irreversible (Sorrells et al., 2018). Therefore, the disability caused by IS is difficult to recover (Wu et al., 2020) and places a heavy burden on patients and society (Feigin et al., 2021). Neuronal death after IS is associated with various cell death mechanisms, including apoptosis, pyroptosis, necrosis, autophagy, ferroptosis, and cuproptosis (Ajoolabady et al., 2021; Cui et al., 2021; Liu et al., 2023b; Luo et al., 2022; Wang et al., 2023). Disulfidptosis is a new cell death mechanism, primarily due to the abnormal accumulation of cystine in cells, inducing disulfide stress, causing the breakdown of the filamentous actin network, and cell death (Joly et al., 2020; Liu X. et al., 2020; Liu et al., 2023a). The link between disulfide death and IS remains unclear. We screened two DE-DRBs, LRPPRC and NDUFA11, using bioinformatic methods. We found that their expressions were significantly downregulated in patients with IS. LRPPRC is a crucial mitochondrial RNA (mtRNA) stabilizing protein (Cui et al., 2019). Loss of LRPPRC can impair mitochondrial bioenergy function and decrease mtRNA stability (Ruzzenente et al., 2012). The NDUFA11 is involved in forming respiratory chain protein complexes in mitochondria, and abnormal expression or structure of NDUFA11 may cause defects in the cellular respiratory chain (Berger et al., 2008). Correlation analysis revealed a correlation between the expression of these two genes. PPI analysis found that LRPPRC and NDUFA11 are indirectly linked via NDUFS1. NDUFS1 forms a protein complex with NDUFA11, NUBPL, and LRPPRC. Previous studies demonstrated that NDUFS1 and NUBPL also form respiratory chain protein complexes within mitochondria (Kimonis et al., 2021; Papa et al., 2009). Combined with these bioinformatic analyses, treating disulfidptosis caused by IS by targeting mitochondria is possible.

With the molecular targets identified, we need to accurately diagnose and treat patients with IS. Cluster analysis can be used to further understand the underlying clinical characteristics and disease mechanisms in patients. Cluster analysis was used to classify patients with IS into two types. Patients with IS in cluster 1 displayed lower LRPPRC and NDUFA11 expression, suggesting that mitochondrial function may be more impaired in these patients. The GO analysis indicated that patients with IS in cluster 1 were mainly immunoreactive. The KEGG result revealed that the signaling pathway of cardiac muscle contraction was active in cluster 1. An active immune response may cause the immune system to release inflammatory mediators, leading to inflammation and worsening of brain damage (Gong et al., 2024). We also observed that the interferon-gamma (IFN-γ)-mediated signaling pathway was significantly active in patients in cluster 1, possibly inhibiting the secretion of the inflammatory factor interleukin-10 (IL-10), upregulating autoimmune function, and reducing susceptibility to infection by pathogenic microorganisms after IS (Prass et al., 2003). Activation of the IFN-γ signaling pathway can decrease IL-10, metanephrine, and normetanephrine in blood circulation, improve lymphocyte function, and reduce bacterial infection (Prass et al., 2003; Yan and Zhang, 2014). Post-IS may cause dysfunction of the brain-heart axis, thereby causing changes in the cardiovascular system (Battaglini et al., 2020). Chen et al. (2021) indicated that IS can cause cardiac atrophy and dysfunction. Mitochondrial damage was more evident in cluster 1 patients, which may cause inflammatory responses, and these patients may develop cardiovascular system dysfunction. Endocrine system functions, including organic acid transport, anion transport, triglyceride sequestering, and aldosterone-regulated sodium reabsorption, were active in cluster 2, suggesting that the endocrine function of these patients may be disturbed. Wang et al. (2020) found that the risk of patients with IS developing acquired hypothyroidism, pituitary dysfunction, or adrenal disorders increased by fold. The KEGG results revealed that the TGF-β signaling pathway was activated in cluster 2. Active TGF-β signaling promotes blood vessel regeneration in the brain, indicating that patients in cluster 2 are more likely to develop collateral circulation and boost their blood supply to the brain. Zhang et al. (2023) found that TGF-β activation stimulates M2 polarization of microglia in the ischemic environment of the brain, potentially regulating the early inflammatory response in the post-ischemic hemisphere and promoting neural function recovery after IS (Feng et al., 2023). In cluster 2 patients, the inflammatory response activates glial cells and may simultaneously affect endocrine and immune functions via the neuroendocrine-immune network. Based on cluster analysis, patients with IS should pay attention to the immune inflammatory response and changes in the cardiovascular and endocrine systems. Therefore, clinicians should pay attention to treating the immune, cardiovascular, and endocrine systems, and the primary disease in patients with IS.

In addition to accurate diagnosis and treatment, predicting the prevalence of IS is important. Recently, ML models have been applied to predict the prevalence of certain diseases. These models consider the relationship between variables using multi-factor analysis, with high reliability (Lai et al., 2022; Qin et al., 2022). Previous studies demonstrated that ML models were employed to predict the risk, diagnosis, therapy, outcome of IS (Lan et al., 2017; Liu et al., 2023c; Qu et al., 2022). We compared the accuracy of the ML models to predict IS and found that the SVM model was more suitable to predict IS. Based on the SVM model, we selected three important genes (*BNIP3, N6AMT1*, and *ICOS*) as predictor genes and used them to construct the SVM prediction model. Then, another dataset was applied to test the accuracy of the SVM model, and the results indicated that the SVM model had good accuracy. *BNIP3*-mediated autophagy is involved in IS pathogenesis (Li et al., 2023a). However, *BNIP3* inhibition protected neurons after IS (Zhang et al., 2022). The roles of *N6AMT1* and *ICOS* in IS remain unclear, and further research is needed.

Finally, we must identify molecular targets associated with disulfidptosis in patients with IS using clinical samples and experimental analysis. The IF results demonstrated that DE-DRBs were present in neurons and cortical regions. The OGD/R and MCAO models were used to further confirm DE-DRB expression levels. Western blot and RT-PCR analyses depicted that LRPPRC expression did not change significantly in the blood of patients with IS, OGD/R, and MCAO groups, while NDUFA11 expression decreased significantly. Evidence from clinical samples and experimental analysis suggests that NDUFSA11 may be considered a DRB in patients with IS. Additionally, we found a decrease in the NDUFS1-NDUFA11 protein complex, suggesting that IS may damage respiratory chain protein complex I in neuronal mitochondria. Damage to the respiratory chain protein complex I of neurons may impair cellular oxygen consumption and increase ROS levels, leading to mitochondrial dysfunction and cell death (Lopez-Fabuel et al., 2016; Qi et al., 2020; Qi et al., 2022). Mitochondrial respiratory chain protein complex I is the most sensitive to IS and the most vulnerable to disruption (Galkin, 2019). Ni et al. (2022) suggested that IS could promote the NADH dehydrogenase in mitochondrial complex I to facilitate reverse electron transport-derived ROS production from complex I and damage the mitochondria.

Administration of glutathione-ethyl ester at the onset of IS prevented the decline of complex I activity and was associated with smaller infarct size and improved neurological outcome (Bahire et al., 2023), suggesting that mitochondrial respiratory chain protein complex I could be a therapeutic target for IS. Network pharmacological analysis has indicated that metformin hydrochloride may be a target drug for NDUFA11. Metformin is the first-line medication to treat type 2 diabetes mellitus in most guidelines; however, the mechanisms underlying its therapeutic action are complex and are still not fully understood. Metformin is a part of adjunct therapy for treating cancer, age-related diseases, and inflammatory diseases (Foretz et al., 2023). Zhang et al. (2023) suggested that metformin is an optimal candidate agent for neural repair in IS. Metformin can expand the neural precursor cell (NPCs) population and drive NPCs toward neuronal diferentiation (Zhang et al., 2023). Additionally, metformin ameliorates inflammation by improving mitochondrial bioenergetics (Bharath et al., 2020). Metformin’s specific therapeutic strategies for IS should be tested in future studies.

However, this study has some limitations. First, there were insufficient clinical samples to validate DE-DRBs, possibly affecting the generalizability of the findings. Additional validation in larger cohorts is necessary. Second, sufficient clinical samples must be used to verify the prediction accuracy of the three most important genes (*BNIP3, N6AMT1*, and *ICOS*). Lastly, the binding site of NDUFS1 to NDUFA11 must be validated to investigate potential mechanisms of damage caused by disulfidptosis to mitochondrial complex I.

## Conclusion

This study identified the role of disulfidptosis in IS and elucidated the underlying molecular mechanisms that contribute to its heterogeneity. The SVM model was the optimal ML model to accurately predict the risk of IS. This study also suggested that NDUFA11 may be a specific biomarker for disulfidptosis, and the NDUFS1-NDUFA11 protein complex was found to change in the IS.

## Data Availability

The original contributions presented in this study are included in this article/Supplementary material, further inquiries can be directed to the corresponding author.

## Footnotes

1
www.ncbi.nlm.nih.gov/geo

2
https://cn.string-db.org/

3
https://www.gsea-msigdb.org/gsea/msigdb/

4
https://dsigdb.tanlab.org/
